# IMPACT OF IMMIGRATION TIMING ON MEDICATION NON-ADHERENCE AND COGNITIVE FUNCTION IN OLDER ADULTS

**DOI:** 10.1093/geroni/igae098.4001

**Published:** 2024-12-31

**Authors:** Yuan Fang, Jayoung Han

**Affiliations:** Binghamton University, State University of New York, Binghamton, New York, United States; Fairleigh Dickinson University School of Pharmacy and Health Sciences, Florham Park, New Jersey, United States

## Abstract

The aging U.S. immigrant population exhibits higher rates of both diagnosed and undiagnosed Alzheimer’s Disease and related dementia compared to U.S.-born residents. Traditional diagnostic methods often fail to detect cognitive decline in immigrants due to cultural and linguistic barriers. Immigration is a multifaceted risk factor affecting cognitive health through acculturation, language proficiency, and healthcare access. This study examines the moderating role of immigration timing in the relationship between medication non-adherence and cognitive function. We analyzed data from the Health and Retirement Study (HRS), focusing on adults >65 years who participated in interviews between 2012-2016 (n=10,869). Cognitive function was assessed using recently developed standardized latent cognitive scores. The relationship between medication non-adherence and cognitive scores was modeled using a moderated moderation model, examining interactions between age at immigration, education, and medication non-adherence, controlling other confounders. Non-immigrants were assigned a high placeholder value for age at immigration, and this variable was rank-normalized for analysis. A sensitivity analysis using the raw age at immigration variable was also conducted. Medication non-adherence, immigrant, and older age at immigration were associated with lower cognitive scores, while higher education was positively associated. Significant negative two-way interactions indicated that immigration status (β=-0.38±0.14, p=0.0006), timing (β=-0.13±0.03, p< 0.0001), and education (β=-0.91±0.03, p=0.0003) moderated the impact of medication non-adherence on cognitive decline. In conclusion, our study showed that medication non-adherence is a significant predictor of cognitive decline, with later immigration worsening cognitive vulnerability, highlighting the moderating effect of immigration age on the relationship between medication non-adherence and cognitive decline.

